# Molecular surveillance of tick‐borne diseases affecting horses in Poland—Own observations

**DOI:** 10.1002/vms3.451

**Published:** 2021-02-23

**Authors:** Oliwier Teodorowski, Marcin Kalinowski, Dagmara Winiarczyk, Radosław Janecki, Stanisław Winiarczyk, Łukasz Adaszek

**Affiliations:** ^1^ Department of Epizootiology and Clinic of Infectious Diseases Faculty of Veterinary Medicine University of Life Sciences Lublin Poland; ^2^ Department and Clinic of Animal Internal Diseases University of Life Sciences Lublin Poland

**Keywords:** horse, PCR, tick‐borne diseases

## Abstract

The purpose of the study was to carry out the molecular surveillance of piroplasmosis, granulocytic anaplasmosis and lyme borreliosis in horses which originated from Poland and exhibited symptoms raising the suspicion of the aforementioned disease units. The presence of *Theileria equi* genetic material was detected in 37 out of 512 examined horses (7.2%), and *Anaplasma phagocytophilum* in 9 (1.8%). The DNA of *Borrelia burgdorferi* was found in 11 out of 204 examined horses (5.4%). The above‐cited results indicate that the problem of tick‐borne diseases affecting horses in Poland is not as significant as in other parts of Europe, however they have to be considered in differential diagnosis of the diseases with lethargy, fever, anaemia and thrombocytopenia.

## Graphical abstract

Tick‐borne diseases such as piroplasmosis, granulocytic anaplasmosis and lyme borreliosis are a recently new problem among horses in Poland. In Poland, piroplasmosis is amongst the most frequent of the tick‐borne diseases diagnosed in horses, whereas granulocytic anaplasmosis is the least frequent. The results of the molecular surveillance of tick‐borne diseases indicate that they do not pose such a significant problem among horses in Poland in comparison to the other parts of Europe. However, they have to be considered in differential diagnosis of the diseases with lethargy, fever, anaemia and thrombocytopenia.

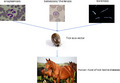

## 1 INTRODUCTION

In Poland, tick‐borne diseases are a relatively new problem. Their occurrence may be related to changing epidemiology of tick‐borne diseases provoking the expansion of ticks—which are disease vectors—to new areas (Dautel et al., 2006; Sréter et al., 2005). Transmission of TBP depends upon a complex arrangement of factors including the presence and abundance of competent vectors, density of competent hosts, climatic factors (temperature, humidity and rainfall) and landscape structure (Matei et al., 2019; Pfäffle et al., 2013).

Among many tick‐borne diseases diagnosed in horses from all over the world, those that stand out in Poland are piroplasmosis (babesiosis/theileriosis), granulocytic anaplasmosis and lyme borreliosis.

Equine piroplasmosis is a serious disease caused by the *Theileria equi* (earlier: *Babesia equi)* and *Babesia caballi* parasites (Mehlhorn & Schein, 1998; Uilenberg, 2006), which are transmitted via ticks of the following genera: *Boophilus*, *Hyalomma, Dermacentor* and *Rhipicephalus*.

The symptoms of babesiosis/theileriosis are diverse—the infected horses may present with icterus, muscle weakness, haemoglobinuria and fever. The protozoa destroy red blood cells (RBCs) causing mechanical damage, which clinically manifests as anaemia. The destruction of red blood cells intensifies due to the antibody opsonisation of RBCs invaded by parasites, and destabilisation of their plasma membrane. Anaemia is concomitant with circulatory disorders, hypoxia, metabolic acidosis and internal organ dysfunction, especially renal and liver failure (Adaszek & Winiarczyk, 2008).

The diagnosis of piroplasmosis is based on the patient history (the presence of a tick on a horse), clinical manifestations, and microscopic examination of blood smears taken from the infected animals (Battsetseg et al., 2002; Rashid et al., 2009). The diagnosis and assessment of the epizootic situation are performed with the use of molecular biology techniques (PCR and sequencing) (Adaszek & Winiarczyk, 2008; Criado et al., 2006).

Granulocytic anaplasmosis is an infectious multiple organ disease occurring with thrombocytopenia. The etiology of the disease in horses includes rickettsia *Anaplasma phagocytophilum* (Bjöersdorff et al., 2002; Dumler et al., 2001; Madigan & Pusterla, 2000).

In Poland, the data regarding the incidence of the disease in animals is very limited. The available references involve cases of anaplasmosis in dogs (Skotarczak et al., 2004), cats (Adaszek et al., 2013) and horses (Adaszek & Winiarczyk, 2011; Adaszek, Winiarczyk, & Łukaszewska, 2009; Adaszek, Winiarczyk, Puchalski, et al., 2009). The genetic material of rickettsia was detected in the blood of fallow deer (*Dama dama*) with no clinical manifestation of the disease (Adaszek, García‐Bocanegra, et al., 2012; Adaszek, Klimiuk, et al., 2012), and specific antibodies against *A. phagocytophilum* were detected in cattle, pigs and dogs (Adaszek & Winiarczyk, 2007; Winiarczyk et al., 2007). There were also two cases of infection caused by *A. phagocytophilum* detected in humans (Tylewska‐Wierzbanowska et al., 2001).

Horses usually become infected through contact with *Ixodes* spp. (Pusterla et al., 1998) and *Rhipicephalus sanguineus* ticks (Inokuma et al., 2000). Anaplasmosis presents non‐specific clinical manifestations. Initially they include lethargy, fatigue and fever. Acute anaplasmosis involves weight loss, bleeding from the mucous membranes, enlargement of the spleen and lymph nodes, arthritis and neurological symptoms such as seizures and paralyses (Reubel et al., 1998). Haematology tests help diagnose thrombocytopenia and anaemia (Goodman et al., 2003; Lotric‐Furlan et al., 2001).

The most simple test used to detect rickettsia in the white blood cells (WBCs) of the infected animals is Giemsa or Diff‐Quick staining of the blood smears (Sells et al., 1976). Due to the fact that it is difficult to detect inclusion bodies in WBCs, microscopic blood smear examination should be followed by positive PCR results (Adaszek et al., 2009; Amusategui et al., 2006; M'ghirbi et al., 2012).

Borreliosis (Lyme disease) is a bacterial disease affecting both animals and humans. It is caused by some members of the spirochete group *Borrelia burgdorferi*, transmitted by *Ixodes* spp. ticks (Post, 1990). Domestic animals, horses and cattle are prone to infection (Burgess et al., 1993; Cohen et al., 1992; Eng et al., 1988; Isogai et al., 1992; Trávnicek et al., 2003).

The course of the disease in horses tends to be asymptomatic. In the clinical form of the infection, the animal is diagnosed with lameness and joint swelling, possibly concomitant with fever (Cohen et al., 1992; Madigan, 1993), uveitis, miscarriage and weight loss (Burgess et al., 1987; Parker & White, 1992). The first step in diagnosing borreliosis is eliminating other disease units which may present similar symptoms to Lyme disease. The next step consists in performing serological (ELISA, Western blott) or molecular (PCR) tests (Stefanciková et al., 2008), as well as assessing the efficiency of tetracycline treatment of the disease (Parker & White, 1992).

There are a few reports on borreliosis in animals in Poland (Adaszek et al., 2008; Adaszek, Winiarczyk, & Łukaszewska, 2009; Adaszek, Winiarczyk, Puchalski, et al., 2009; Adaszek, Winiarczyk, & Górna; 2010; Adaszek, Winiarczyk, Puchalski, et al., 2010). The presence of spirochaete was proven in *Ixodes ricinus* tick bodies, taken from different areas in Poland (Cisak et al., 2005; Zygner et al., 2008). Serological testing performed on horse populations in 12 regions indicated that over 25% of the examined animals had contact with spirochaete (Stefanciková et al., 2008).

The purpose of the study was to carry out molecular surveillance of piroplasmosis, granulocytic anaplasmosis and lyme borreliosis in horses which originated from Poland and exhibited symptoms raising the suspicion of these diseases.

## 2 MATERIALS AND METHODS

The surveillance was carried out between 2013 and 2020. It included 512 horses (362 mares and 150 stallions), aged 2–16 years. A total of 308 animals (group 1) underwent molecular diagnosis towards anaplasmosis and piroplasmosis, whereas the remaining 204 cases (group 2) underwent testing for anaplasmosis, piroplasmosis and borreliosis. In the past, all horses had contact with ticks which resulted in symptoms suggesting babesiosis, anaplasmosis or borreliosis. The animals from the first group presented with lethargy, anaemia and their haematology testing showed thrombocytopenia, whereas the second group presented with lethargy, fever, anaemia and thrombocytopenia concomitant with movement difficulties: lameness or stiff gait and swollen limb joints.

All animals involved in the study had their blood examined via molecular testing for piroplasmosis and anaplasmosis. Additionally, examinations of the animals from the second group included synovial fluid molecular testing for borreliosis. In all cases material for molecular testing was taken within 7 days after the first clinical symptoms appeared.

DNA was obtained from the samples using a commercial DNA Genomic kit (“A&A Biotechnology” Gdańsk, Poland) following the manufacturer's instructions. PCR analyses were performed as described previously to detect *Anaplasma* spp. (Adaszek & Winiarczyk, 2011), *Babesia/Theileria* spp. (Adaszek et al., 2011) and *B. burgdorferi* s.l. (Lee et al., 2019). DNA of *A. phagocytophilum*. and *B. burgdorferi* s.l. were obtained from the National Reference Center for Borrelia of the Max von Pettenkofer Institute, and DNA of *Babesia* from previous studies (Adaszek et al., 2011), both of which were used as positive controls. PCR amplification was performed using a programmable thermocycler (“Biometra”, Goettingen, Germany). The size of each PCR product was analysed by electrophoresis in a 1.5% agarose gel stained with ethidium bromide.

The identification of pathogens was achieved by sequencing PCR products. Purification was performed using QIAquick spin columns (“Qiagen”, Hilden, Germany) and eluted in 50 μl of Tris 10 mM, pH 7.6. DNA sequencing was performed on both strands using the same primers employed for PCR at the DNA Sequencing and Synthesis Service of the Institute of Biochemistry and Biophysics, Polish Academy of Sciences, Warsaw, Poland.

DNA sequences were assembled and edited using SeqMan (DNAStar, Lasergene, USA) and MegAlign (DNAStar, Lasergene, USA), with alignments to the published *A. phagocytophilum* 16S rRNA gene GU183908, *Theileria equi* 18S RNA gene DQ287951, and *B. burgdorferi* s.l. 16S rRNA gene DQ111061.

## 3 RESULTS

In the first group, the presence of genetic material of piroplasmas was detected in 23 blood samples, whereas the DNA of *Anaplasma* was present in 7 samples. All protozoa detected in the blood samples of horses from the first group belonged to the *Theileria equi* species (similarity to the DQ287951 sequence: 99.3%–100%), whereas all strains of rickettsia were identified as *Anaplasma phagocytophilum* (similarity to the GU183908 sequence: 99.6%–100%). There were no cases of mixed infections in this group.

In the second group, the presence of *Theileria equi* DNA was detected in 14 horses (similarity to the DQ287951 sequence: 99.5%–99.8%), *Anaplasma phagocytophilum* in 2 animals (similarity: 99.6%–100%), and *Borrelia burgdorferi* in the synovial fluid of 11 horses (similarity to the DQ111061 sequence 97.2%–98.8%). There were 7 cases of mixed infections associated with *Theileria/Borrelia*. The sequences of *A*. *phagocytophilum, Theileria equi*, and *Borrelia burgdorferi* obtained in the present study were deposited in the GenBank database (GenBank Accession Numbers: MW375665, MW375666‐ MW375668, and MW375712, MW375733, MW375608 respectively).

Table 1 presents the distribution of prevalence of aforementioned tick‐borne pathogens from the particular years of the study.

**TABLE 1:** Distribution of prevalence of selected tick‐borne pathogens in particular years of the study

Year of the study	Positive animals (%)			Number examined/number of horses examined only for *Borrelia*
	*Anaplasma phagocytophilum*	*Babesia/Theileria* spp.	*Borrelia burgdorferi* sensu lato^a^	
2013	1 (2.8)	7 (19.4)	1 (25)	36/4
2014	0 (0.0)	0 (0.0)	0 (0.0)	25/3
2015	2 (4.3)	4 (8.5)	0 (0.0)	47/11
2016	0 (0.0)	4 (6.5)	2 (7.7)	62/26
2017	0 (0.0)	0 (0.0)	0 (0.0)	59/21
2018	1 (1.1)	5 (5.7)	1 (3.1)	88/32
2019	2 (1.8)	7 (6.3)	4 (6.0)	112/66
2020	3 (3.6)	10 (12.1)	3 (7.3)	83/41
Total	9 (1.8)	37 (7.2)	11 (5.4)	512/204

## 4 DISCUSSION

The presence of *Theileria equi* DNA was detected in 37 out of 512 examined horses (7.2%), and *Anaplasma phagocytophilum* in 9 (1.8%). The DNA of *Borrelia burgdorferi* was found in 11 out of 204 examined horses (5.4%).

Having compared the incidence of the studied diseases in Polish horses and those originating from other parts of Europe, it can be concluded that Poland is not an area where there is a high risk of their occurrence. Until recently, diseases such as anaplasmosis, theilerosis or borreliosis were considered as exotic, and were diagnosed sporadically in the horse population in our country. The increase in the incidence of these diseases is probably due to: improving of the diagnostic methods, changes/fluctuations in the microhabitat, and general environmental changes—a warming trend being observed in many regions of the world, including central Europe (Altizer et al. 2013).

Climate, as well as microhabitat changes, host abundance, and social factors may explain the upsurge of diseases transmitted by ticks to animals, as well as humans (Alkishe et al., 2017; Estrada‐Peña et al., 2012; Ostfeld & Brunner, 2015).

Herein we focused on tick‐borne pathogens (*Borrelia burgdorferi* s.l.—Lyme disease), *Anaplasma phagocytophilum*—granulocytic anaplasmosis, and *Theileria equi ‐*theilerosis), that affect horses with epidemic potential.

According to studies which involved the equidae population in Spain, the presence of *T. equi* genetic material was detected in as many as 20% of animals with no clinical manifestations, which reflects the persistence of babesiosis/theileriosis in that region. This is proven by serological testing results, which showed the presence of specific protozoa antibodies in over 58% of stud farms located in southern Spain (García‐Bocanegra et al., 2013). A slightly smaller percentage of horses with piroplasma antibodies (34.6%) was recorded in Turkey, in the Black Sea basin area (Acici et al., 2008). A relatively high seroprevalence was observed among horses in Hungarian stud farms, where the presence of specific protozoa antibodies was detected in animals from 17 of 27 examined farms (67.9%) (Farkas et al., 2013).

In the northern region of Italy, the percentage of horses with specific antibodies against *T. equi* and *B. caballi* amounted to 8.5%. Through the PCR technique, the presence of piroplasma genetic material was detected in the blood of 33% of seroreagentsseroreagents, which proves the considerable incidence of protozoa infections in that area too (Grandi et al., 2011).

In accordance with the study, granulocytic anaplasmosis was detected in only 9 horses, which makes it the least frequently diagnosed unit among the three diseases under surveillance. Thus, it confirms that the central and eastern regions of Europe are not an endemic area of the disease. Similar observations were made in the Czech Republic by Jahn et al. (2010), who in 2002–2008 diagnosed only 12 cases of granulocytic anaplasmosis in horses and suspected five other cases.

Also, there is a smaller incidence of contact with rickettsia than, e.g. piroplasma among horses in France. According to the studies carried out by Leblond et al. (2005), serological testing detected the presence of specific antibodies for *A. phagocytophilum* in 11.3% of horses, while antibodies for *T. equi* and *B. caballi* were present in 64.4% and 19.7% of horses, respectively.

The available references offer limited data regarding molecular biology surveillance of borreliosis among horses in Europe (Veronesi et al., 2012). Techniques such as polymerase chain reaction and possible sequencing of the amplified product were used in the diagnosis of individual cases of Lyme disease and in the identification of its etiological factor (Hulinska et al., 2002; James et al., 2010; Sears et al., 2012). The risk of developing borreliosis by horses in a given area is usually evaluated upon the screening of serological test results. The percentage of unaffected horses positive for specific antibodies against spirochete amounted to 24.3% in Italy (Ebani et al., 2012), 29% in Denmark (Hansen et al., 2010), and only 6% in Turkey (Bhide et al., 2008). Serological surveillance carried out among the horse population in Poland showed the presence of specific antibodies against *B. burgdorferi* sensu lato in 25.6% of the examined individuals (Stefanciková et al., 2008). It should be stressed that the cited studies were performed with the use of various serological tests, which did not involve standardised antigens but complete cells or isolated proteins from bacteria, and this undoubtedly affected the results to some extent.

In‐house examinations detected 7 cases of mixed infections associated with *Theileria/Borrelia*. It is not surprising to find two pathogens in a horse's body simultaneously. Both borreliosis and piroplasmosis may be transmitted by the same species of ticks: *Dermacentor* and *Rhipicephalus* (Baptista et al., 2004; Kerber et al., 2009; Nijhof et al., 2007), therefore, the possibility of developing mixed infections should always be taken into account when tick‐borne diseases are suspected. Proper recognition of all pathogens is also key to therapeutic success. It is important to remember that in the case of incomplete diagnosis, the treatment may be ineffective.

## CONFLICT OF INTEREST

The authors declare that they have no conflict of interest.

## AUTHOR CONTRIBUTION

**Oliwier Teodorowski:** Conceptualization; Investigation. **Marcin Kalinowski:** Formal analysis; Methodology. **Dagmara Winiarczyk:** Data curation; Investigation. **Radosław Janecki:** Data curation; Methodology. **Stanisław Winiarczyk:** Conceptualization; Resources; Supervision. **Łukasz Adaszek:** Conceptualization; Project administration; Writing‐original draft.

### PEER REVIEW

The peer review history for this article is available at https://publons.com/publon/10.1002/vms3.451.
